# Histopathologic Findings in Three Dogs With Metastatic Carcinoma Presenting With Bronchovascular Bundle Thickening on Computed Tomography

**DOI:** 10.1111/vru.70120

**Published:** 2025-12-08

**Authors:** Robert Wise, Maria Mulvihill, Jennifer Reetz, Bianca Pfisterer, Wilfried Mai

**Affiliations:** ^1^ Department of Clinical Sciences and Advanced Medicine School of Veterinary Medicine Section of Radiology University of Pennsylvania Philadelphia Pennsylvania USA; ^2^ Department of Pathobiology New Bolton Center University of Pennsylvania School of Veterinary Medicine Kennett Square Pennsylvania USA

**Keywords:** aerogenous metastasis, lymphangitic carcinomatosis, peribronchovascular thickening, pulmonary carcinoma, septal metastasis

## Abstract

Carcinoma metastasis to the lungs can occur via multiple pathways, most notably via hematogenous, lymphangitic, or aerogenous routes. Computed tomographic (CT) characteristics of these metastatic pathways can vary, with only sparse reports of bronchovascular bundle thickening (BVBT) associated with metastatic carcinoma. In this retrospective cross‐sectional study, three dogs with metastatic carcinoma and BVBT on CT that had corresponding histopathological sections at the site of BVBT were reviewed. These cases showed expansion of the peribronchial/peribronchiolar interstitium with islands of neoplastic cells within the stroma and either lymphatic infiltration alone or hematogenous and lymphatic infiltration combined (lymphovascular), along with neoplastic cells in the airways and/or septal lining of the alveoli. On the basis of this study, BVBT on CT in dogs can be seen with metastatic carcinoma, associated with peribronchial/peribronchiolar lymphovascular and aerogenous metastases. Additional studies are needed to characterize each metastatic pathway and further correlate histopathological findings with CT appearance.

## 1 Introduction

Thoracic computed tomography (CT) is increasingly used as a staging tool in canine and feline cancer patients. On radiographs or CT, pulmonary metastases typically appear as nodular soft tissue opacities of variable sizes. Variants in the presentation of pulmonary metastases have rarely been reported. In four dogs with pulmonary metastatic disease from mammary carcinoma, the radiographic changes were characterized by a generalized severe mixed pulmonary pattern, including a marked unstructured interstitial and cranioventral alveolar pattern [1]. Histologically, there were neoplastic cells in the pulmonary arteries and small capillaries of the alveolar septa and in the bronchial lymphatics and interstitium. This histologic appearance was reportedly consistent with diffuse lymphangitic metastasis (lymphangitic carcinomatosis), as is seen in humans. However, the authors felt that the bulk of the neoplastic infiltrate was in the alveolar septal capillaries, which do not contain lymphatic vessels. As a result, they labeled this condition as pulmonary alveolar septal metastasis [1]. On CT, similar patterns of wedge‐shaped to amorphous ground glass opacity and consolidation have been occasionally described with pulmonary metastatic disease in dogs and humans [2, 3, 4, 5]. A radiographic or CT pattern of bronchial thickening has also been anecdotally described in textbooks [2, 4, 6, 7].

In our hospital, we have observed some dogs with striking bronchovascular bundle thickening (BVBT) on CT, primarily in those with confirmed or suspected primary pulmonary carcinoma that were imaged for surgical planning and staging. The bronchovascular bundle comprises the bronchi and adjacent interstitium along with pulmonary arteries, veins, and lymphatics, extending radially from the hila of the lungs [4, 6]. BVBT on CT scans refers to the appearance of branching lines of variable thickness along a peribronchovascular distribution, creating the appearance of bronchial thickening, similar to pulmonary lymphangitic carcinomatosis seen in humans [8, 9, 10, 11, 12]. This metastatic invasion of the interstitial lymphatics may occur through different pathways, including antegrade invasion of lymphatics through the pleura or diaphragm, retrograde spread from metastatic hilar lymph nodes, and/or hematogenous spread with direct neoplastic invasion from blood vessels into the adjacent lymphatic vessels and interstitium [8, 9, 13, 14, 15, 16]. Additional CT features of lymphangitic carcinomatosis in humans include interlobular septal thickening and reticulonodular structures [8, 9, 10, 11, 12, 14].

The other pathways for pulmonary metastatic disease described in humans are hematogenous (most common), aerogenous (also known as endobronchial), pleural, and direct chest invasion [5, 8, 9, 14, 17]. The aerogenous metastatic pathway is a newer concept, characterized as intra‐airway seeding [5, 8, 14, 17]. It is postulated that cancer cells along the alveolar septa at the primary site detach from the basal membrane, spread through the airways, and reattach and grow along the alveolar septa away from the primary lesion. CT features of aerogenous spread in humans include centrilobular with branching opacities (such as with tree‐in‐bud nodules), typically with ill‐defined margins and ground‐glass attenuation. In dogs, the primary described pathways are the hematogenous and lymphatic routes. Additionally, aerogenous metastasis is thought to occur in dogs with primary pulmonary carcinoma [18].

The purpose of this study was to evaluate the histopathological findings and metastatic pathways in dogs with confirmed carcinoma and BVBT on CT. Our goal was to provide a direct correlation of BVBT on CT with histopathological BVBT to determine the distribution of neoplastic cells, for example, peribronchial/peribronchiolar lymphatics, pulmonary arteries/veins, alveolar septal capillaries, and/or airways.

## 2 Methods

This was a retrospective, descriptive cross‐sectional study with histopathological evaluation. All owners had signed consent forms to use patient data at admission to the hospital, and all animals were imaged for clinical reasons; as a result, no additional approval was required from the university's Institutional Animal Care and Use Committee. With the hospital director's approval, imaging archives from a single veterinary teaching hospital (University of Pennsylvania, Ryan Veterinary Hospital) were searched for dogs with computed tomography findings of pulmonary mass, pulmonary metastasis, lymphangitic carcinoma, lymphangitic metastasis, pulmonary neoplasia, carcinoma, and septal metastasis during the period from 2007 to 2024. Inclusion criteria included dogs with a pulmonary mass with evidence of BVBT, which extended immediately and/or distantly from the mass, and histopathological lung examination, via surgical biopsy and/or necropsy, that exhibited evidence of BVBT. All decisions for subject inclusion or exclusion were made on the basis of a consensus between a radiology resident (M.M.) and American College of Veterinary Radiology (ACVR)‐certified radiologists (J.A.R. and W.M.).

For dogs that met the initial inclusion criteria, two radiology residents (M.M. and R.W.) retrieved and reviewed medical records, CT scans, and histopathological findings from the hospital archive. Information collected from the medical record, in addition to the histological results, included age, sex, breed, weight (kg), and clinical signs.

CT images of the cases that met the inclusion criteria were reviewed using a standard DICOM viewing software (Sectra IDS7) by radiology residents (M.M. and R.W.) and then reviewed by ACVR‐certified radiologists (J.A.R. and W.M.) using both lung windows (width, 1800 Hounsfield units [HU]; level, −585 HU) and soft tissue windows (width, 400 HU; level, 40 HU). The information recorded included the following: (1) presence and location of the pulmonary mass(es) and (2) location and distribution of BVBT, including unilobar versus multilobar, ipsilateral versus contralateral to a mass (denoted as bilateral if BVBT was both ipsilateral and contralateral to a mass), adjacent to or in the same lobe as a mass, and/or in other lobes distant to a mass.

Thoracic CT studies were performed with a 16‐row detector CT scanner (Bright Speed, GE Healthcare, Milwaukee, WI; Philips Gemini TF Big Bore Scanner, Philips Medical Systems, the Netherlands), except for one patient, who was scanned with a 64‐row detector CT scanner (Revolution Maxima, GE Healthcare, Waukesha, WI). All patients were under general anesthesia and positioned in sternal recumbency, with either a breath‐holding technique (using positive pressure ventilation) or hyperventilation prior to the scan, with imaging during apnea. The following scan settings were used: helical scan mode; slice thickness, 0.625, 1.25, or 2.5 mm; helical pitch, 0.8–1; matrix dimensions, 512 × 512 or 508 × 508; varied field of views; and low‐frequency and high‐frequency reconstruction algorithms. Pre‐contrast series and an approximately 1–2 min post‐contrast administration series were available for all cases. For the contrast‐enhanced CT scan, a nonionic iodinated contrast medium (Iohexol 350 mgl/mL, Omnipaque 350, GE Healthcare, Princeton, NJ) was administered (700–770 mgl/kg body weight) in a cephalic vein either manually or with a power injector at a flow rate of 2–2.2 mL/s.

Slides for specific affected lung lobe regions (as determined by the CT images and gross pathologic findings) were reevaluated by an American College of Veterinary Pathology certified pathologist (B.P.). The histopathological primary diagnosis and the presence of metastasis (hematogenous, lymphangitic, and/or aerogenous) were evaluated in each case. Hematogenous metastasis, also known as vascular invasion, was defined as neoplastic cells within peribronchial/peribronchiolar arterial and venous vessels, as well as within the capillaries of alveolar septa. Lymphangitic metastasis was defined as neoplastic cells within peribronchial/peribronchiolar lymphatic vessels. Lymphovascular invasion was defined as neoplastic cells within both hematogenous and lymphatic vessels. Aerogenous metastasis was defined as neoplastic cells within the bronchi/bronchioles lumens, alveoli, and/or septal lining of the alveoli. The prevalence of histological location and metastatic pathway was recorded (hematogenous, lymphangitic, lymphovascular, and aerogenous).

## 3 Results

Initially, over 1000 CTs were reviewed, and 138 met the initial CT inclusion criteria, having at least one pulmonary mass and BVBT. Of the 138 CTs, 14 cases had pulmonary histopathology. Eleven of these cases were excluded after a review of the histopathologic results showed missing/inadequate samples, or the sample location was not definitively taken from regions of BVBT as seen on CT, which did not allow direct radiological and histopathological correlation. Therefore, the final sample for analysis consisted of two female spayed and one male castrated dog. Ages ranged from 8 to 11 years old (median 10 years). Weights ranged from 14.5 to 30.9 kg (median 15.5 kg). Breeds were mixed‐breed dog, beagle, and cocker spaniel. Presenting clinical signs included coughing, lethargy, polydipsia, hyporexia, and collapse.

**FIGURE 1:**
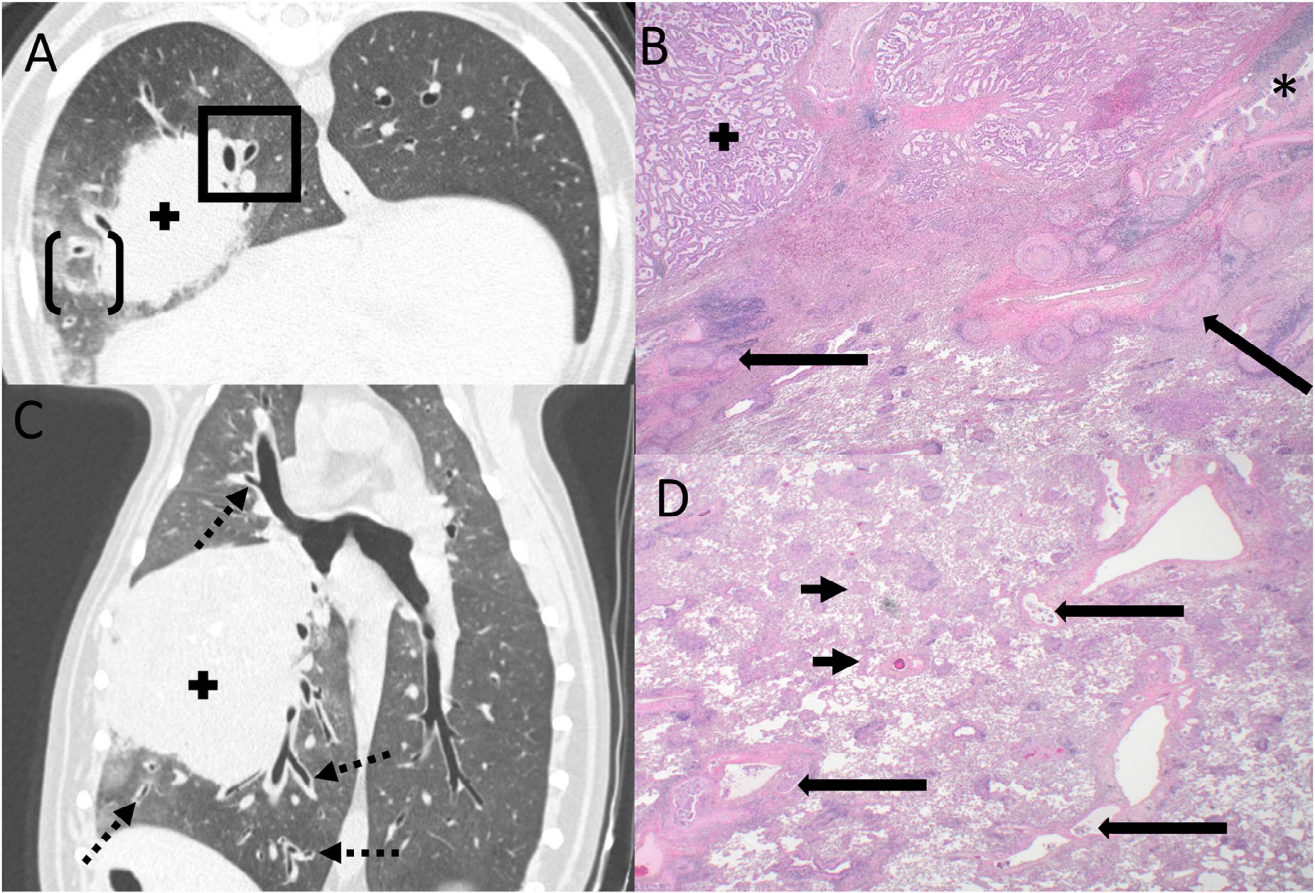
Transverse (A) and dorsal plane reformatted (C) CT images and corresponding histopathologic images at 20× magnification (B and D) of Case 2. (A) Bronchovascular bundle thickening (center of the black square) in the right caudal lung lobe adjacent to a primary pulmonary adenocarcinoma (+). Ground glass opacity, in addition to BVBT, along the ventral periphery of the mass, is present (black open brackets). (B) Histological sample taken from the area in the black square in (A), including the mass (+) and adjacent bronchovascular bundle thickening, demonstrating a bronchus (*) surrounded by collagen and islands of neoplastic cells (long black arrows). (C) Multilobar ipsilateral bronchovascular bundle thickening is present in the right caudal and right cranial lung lobes, adjacent to and extending distant to the mass (dashed arrows). Bronchovascular bundles are normal in the left lung. (D) Histological sample taken from the area in the black open bracket in (A) demonstrating primarily vascular metastases (long black arrows) and septal thickening (short black arrows). CT images: window width 1500, window level −700.

**FIGURE 2:**
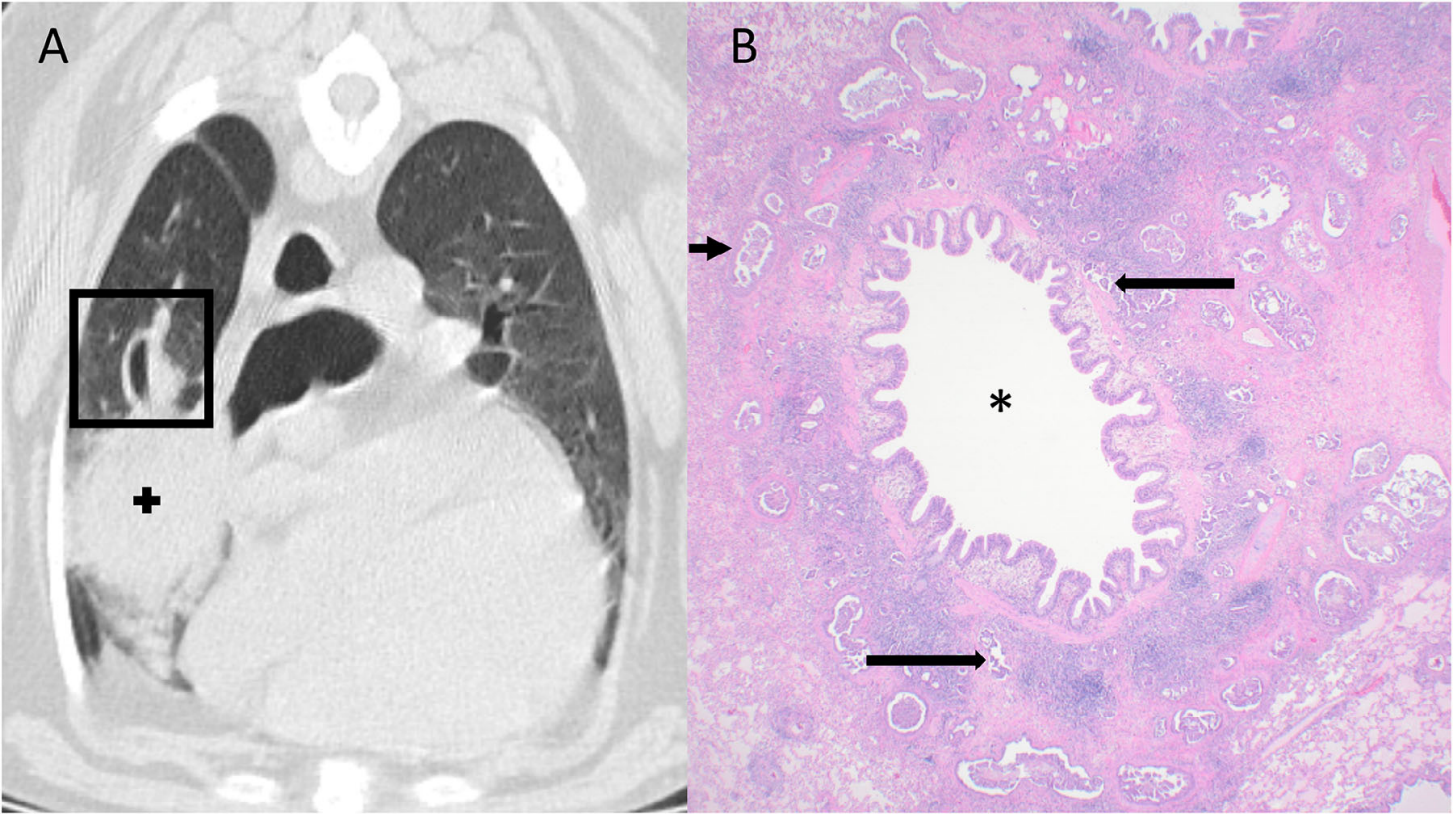
Transverse CT image (A) and corresponding histopathologic image at 20× magnification (B) of Case 3. (A) Bronchovascular bundle thickening is present in the right cranial lung lobe (center of the black square) adjacent to a primary pulmonary carcinoma (+). Window width 1700, window level −600. (B) Corresponding histopathological sample shows changes located around the bronchus (*), including peribronchial thickening with increased collagen and islands of neoplastic cells (short black arrow) and numerous small lymphatics filled with neoplastic cells (long black arrows).

All three dogs had one primary lung mass: one in the right caudal lung lobe, one in the right cranial lung lobe, and one in the caudal segment of the left cranial lung lobe. In two dogs, BVBT was ipsilateral and multilobar, including within the lobe containing the primary mass (Figure 1A,C). In the third dog, the BVBT was unilobar and located in the lobe with the lung mass (Figure 2A). All three dogs had biopsies performed after lobectomy of a single pulmonary mass, with histopathologic samples taken from the mass and some of the adjacent lung tissue. All three dogs were diagnosed with primary pulmonary carcinomas (one adenocarcinoma and two unspecified carcinomas). These findings are summarized in Table 1.

**TABLE 1:** Pulmonary carcinoma type and location, pulmonary computed tomographic (CT) changes, including bronchovascular bundle thickening (BVBT) location and distribution, lung sampling location, and histopathologic changes.

Case	Type of neoplasia	Pulmonary neoplasia location (CT, gross, and histopathology)	BVBT location and distribution (CT)^a^	Lung lobe sample location (histopathology)	Location of neoplastic cells (histopathology)	Metastatic pathway type
Case 1	Pulmonary carcinoma	Single mass—caudal segment of the left cranial lung lobe	Unilobar and ipsilateral	Left cranial	Peribronchiolar interstitium Alveolar lumen	Lymphovascular Aerogenous
Case 2	Pulmonary adenocarcinoma	Single mass—right caudal lung lobe	Multilobar and ipsilateral	Right caudal	Peribronchiolar interstitium Peribronchial interstitium Alveolar lumen Septa	Lymphovascular Aerogenous
Case 3	Pulmonary carcinoma	Single mass—right cranial lung lobe	Multilobar and ipsilateral	Right cranial	Peribronchiolar interstitium Peribronchial interstitium Alveolar lumen Septa	Lymphangitic Aerogenous

^a^ BVBT was present in the lobe with the mass (whether unilobar or multilobar BVBT).

Histopathology showed primarily lymphovascular invasion in Cases 1 and 2 and lymphangitic invasion in Case 3. All three cases had aerogenous metastasis. More specifically, Case 1 showed severe bronchial wall edema and hemorrhage with neoplastic cells adjacent to affected bronchioles, especially near subpleural areas. Aerogenous invasion was noted adjacent to affected bronchioles but not bronchi in this case. Case 2 showed severe expansion of the peribronchial and peribronchiolar interstitium with islands of neoplastic cells surrounded by desmoplasia in addition to islands of neoplastic cells within lymphatic and/or blood vessels (Figure 1B,D). Case 2 also had neoplastic cells within alveoli and septa adjacent to affected bronchi and bronchioles (aerogenous). Case 3 showed peribronchial thickening with increased collagen and islands of neoplastic cells, as well as neoplastic cells with lymphatics (Figure 2B). This case had minimal neoplastic cells within septa or alveoli distant from the peribronchial/peribronchiolar thickening (scarce aerogenous).

## 4 Discussion

The results of this study demonstrated that BVBT on CT may be associated with metastatic carcinoma. This study also described the histological distribution of neoplastic cells and the various corresponding metastatic pathways that can occur with metastatic carcinoma, including lymphangitic, hematogenous, and aerogenous metastasis.

In the three dogs with distinct histopathologic findings corresponding to BVBT on CT, the BVBT represented neoplastic infiltration of the peribronchial/peribronchiolar lymphatic vessels, blood vessels, or both (i.e., lymphovascular) in addition to expansion of the interstitium with neoplastic cells surrounded by desmoplasia. Infiltration of the lymphatic vessels (and adjacent peribronchial/peribronchiolar interstitium) correlates with lymphangitic carcinomatosis in humans, which causes BVBT on CT [8, 9, 10, 11, 12]. However, we also found neoplastic infiltration of the peribronchial/peribronchiolar blood vessels (hematogenous metastases) and within the bronchi/bronchioles lumens, alveoli, and/or septal lining of the alveoli (aerogenous metastasis), and therefore, in this sample, the presence of BVBT on CT in dogs is not necessarily specific to a single metastatic pathway. This was also described in several dogs in a prior study, where some typical CT characteristics of lymphangitic metastasis, as described in humans, correlated better with hematogenous spread on histopathology [2]. Interestingly, however, one potential way for neoplastic cells to reach the lymphatics is through an initial hematogenous spread, with secondary invasion of the lymphatic vessels [8, 15, 16]. Regardless, the potential utilization of the terminology lymphangitic carcinomatosis for BVBT in dogs, as is used in humans, may not be entirely appropriate, as it may not be an all‐encompassing term for the potential metastatic pathways.

Aerogenous metastases were seen in all three cases. This is not a well‐described entity in dogs and is still a newer concept in humans [5, 8, 14, 17]. CT characteristics of aerogenous metastases described in humans are centrilobular, typically ill‐defined nodules with branching opacities (tree‐in‐bud) and ground glass attenuation affecting predominantly or exclusively the air space [14, 17]. Recently, the term “spread through air space” or STAS was used to describe aerogenous intrapulmonary metastasis of pulmonary adenocarcinoma, where ground glass nodules along the bronchioles were identified on CT, and histopathology showed clusters of cancer cells on the bronchiolar mucosal surface or in the air space, without involvement of the regional lymphovascular structures, which supported an airborne mechanism of spread [17]. In our three dogs with BVBT and matching histopathological sections, one of the cases had neoplastic cells in alveoli and alveolar septa adjacent to the bronchi and larger bronchioles, which could potentially have contributed to the BVBT on CT. The other two cases had aerogenous metastasis distant from the BVBT. Further investigation of the aerogenous metastatic pathway and its potential appearance on CT in veterinary medicine is warranted.

Nonneoplastic diseases of the bronchovascular bundle have also been reported in humans (e.g., tuberculosis, nontuberculous mycobacterial or other granulomatous infection, asthma, and pulmonary edema or hemorrhage) and in animals (e.g., infectious bronchitis and immune‐mediated bronchitis/asthma) [3, 4]. Future studies are needed to further evaluate possible etiologies for BVBT, including other neoplastic and nonneoplastic causes.

This study's major limitations were its retrospective nature and small sample size. Although we identified a larger number of dogs with a BVBT pattern on CT, only three cases had a biopsy sample from the margin of the pulmonary mass, including the BVBT, which allowed for a direct imaging/pathology correlation. Larger multi‐institutional studies may be useful to provide further evidence on the underlying pathology of this pattern in dogs with pulmonary metastases.

In conclusion, this study supports that BVBT on CT in dogs can be seen with metastatic carcinoma and is associated with peribronchial/peribronchiolar lymphovascular neoplastic infiltration in addition to neoplastic cells in the airways and/or septal lining of the alveoli. On the basis of these findings, the term “lymphangitic carcinomatosis” used in humans may not be the most appropriate terminology for this imaging feature, as it may not encompass all the possible metastatic pathways. This study also introduced the concept that aerogenous metastasis is a possibility in dogs. Future studies with histopathological correlation are needed in veterinary medicine to further investigate specific CT characteristics of the various metastatic pathways.

## Author Contributions

Conception and design: Maria Mulvihill, Robert Wise, Jennifer Reetz, and Wilfried Mai. Acquisition of data: Maria Mulvihill, Robert Wise, Jennifer Reetz, Bianca Pfisterer, and Wilfried Mai. Analysis and interpretation of data: Robert Wise, Maria Mulvihill, Jennifer Reetz, Bianca Pfisterer, and Wilfried Mai. Drafting the article: Robert Wise, Jennifer Reetz, Bianca Pfisterer, and Wilfried Mai. Reviewing article for intellectual content: Robert Wise, Maria Mulvihill, Jennifer Reetz, Bianca Pfisterer, and Wilfried Mai. Final approval of the completed article: Robert Wise, Maria Mulvihill, Jennifer Reetz, Bianca Pfisterer, and Wilfried Mai. Agreement to be accountable for all aspects of the work in ensuring that questions related to the accuracy or integrity of any part of the work are appropriately investigated and resolved: Robert Wise, Maria Mulvihill, Jennifer Reetz, Bianca Pfisterer, and Wilfried Mai.

## Disclosure

No previous presentation or publication disclosure. The STROBE‐VET checklist was used.

## Conflicts of Interest

The authors declare no conflicts of interest.

## Data Availability

The data used in the study are available from the corresponding author upon reasonable request.
